# Imaging Techniques to Differentiate Benign Testicular Masses from Germ Cell Tumors

**DOI:** 10.1007/s11934-023-01172-7

**Published:** 2023-06-27

**Authors:** Ava Saidian, Aditya Bagrodia

**Affiliations:** 1 Department of Urology, University of California San Diego Health, 9400 Campus Point Drive #7897, 92093-7897 La Jolla, CA USA; 2 Moores Cancer Center, San Diego, CA USA

**Keywords:** Testicular cancer, Testicular imaging, Ultrasound, Magnetic resonance imaging

## Abstract

**Purpose of Review:**

To discuss role of different diagnostic imaging modalities in differentiation of benign testicular masses from seminomatous germ cell tumors (SGCTs) and non-seminomatous GCTs (NSGCTs).

**Recent Findings:**

New modalities of ultrasonography, including contrast enhancement and shear wave elastography, may help differentiate between benign and malignant intratesticular lesions.

**Summary:**

Ultrasonography remains the recommended imaging modality for initial evaluation of testicular masses. However, MRI can be used to better define equivocal testicular lesions on US.

## Introduction

Testicular germ cell tumors (GCTs) are a rare malignancy with peak incidence in men aged 20 to 34 years [1]. Disease staging is critical in deciding treatment choice and sequence of treatments as more than 90% of testicular GCTs are considered curable. Testicular cancer staging includes a combination of tumor histopathology, assessment of lymph nodes and metastases on diagnostic imaging, and serum tumor markers [2••]. A scrotal mass with suspicion for testicular GCT is evaluated with physical exam, testicular ultrasound, and serum tumor markers. Accurate characterization of scrotal lesions is important as the management can range from observation to surgical resection. A missed diagnosis of a testicular germ cell tumor can lead to delays in diagnosis, advanced stage at presentation, treatment intensification, and worse clinical outcomes. Conversely, unnecessary orchiectomy for benign scrotal pathology can negatively affect androgen function, fertility parameters, and body image. Accordingly, it is paramount that we can distinguish benign from malignant testicular lesions.

This article reviews the role of different diagnostic imaging modalities in differentiation of benign testicular masses from seminomatous (SGCTs) and non-seminomatous GCTs (NSGCTs).

## Ultrasonography (US)

B-mode high-frequency (greater than or equal to 10 MHz) grayscale scrotal sonography performed with a linear-array transducer is the initial imaging modality used to evaluate testicular masses suspicious for malignancy. GCTs are often intratesticular masses, and US can accurately distinguish between intratesticular and extratesticular lesions [3]. SGCTs appear hyperechoic and homogenous compared with healthy testicular tissues. They may be lobulated or multinodular and rarely have calcifications (30%) or cystic spaces (10%) [4]. NSGTs often appear as multicomponent masses on grayscale sonography and can be solid or solid-cystic lesions [5•]. Color-coded duplex sonography can be used to analyze the vascularization of intratesticular masses with malignant lesions often demonstrating increased vascularity compared with background testis tissue [4]. Grayscale US (combined with clinical presentation) can be used to distinguish between GCTs and benign testicular masses such as testicular hematoma, epidermoid cyst, adrenal rests, splenogonadal fusion, and sex-cord stromal tumors (Table 1).

**Table 1:** Imaging features of benign masses on US and MRI

Lesion	Clinical feature	Imaging features US	Imaging features MRI
Testicular hematoma	Recent trauma	Avascular, iso- to hyperechoic, become hypoechoic over time	Hyperintense initially on T1, hypointense rim on T2 over time; no contrast enhancement
Epidermoid cyst	Painless mass	Well-defined rounded lesions with onion ring internal pattern of echoes	Absence of contrast enhancement; T1 hypointensity, T2 hyperintensity with hypointense rim
Adrenal rest	Often in patients with CAH	Hypoechoic, bilateral	Contrast enhancement, low T2 signal intensity
Splenogonadal fusion	Painless mass	Splenic tissue is hypoechoic, often with central vascular pattern with vessels branching towards periphery	May show continuous or discontinuous relation between ectopic splenic tissue and gonad
Sex-cord stromal	Precocious puberty, gynecomastia	Focal hypoechoic mass	Low T2 signal intensity; mild contrast enhancement
Lipoma	Painless mass	Hypoechoic, homogenous	High T1 signal intensity
Testicular cyst	Non-palpable, discovered incidentally	Anechoic with posterior acoustic enhancement; well-marginated	Lack contrast enhancement or solid components

Shear wave elastography (SWE) is an US modality that provides quantitative color-coded maps of tissues stiffness that are displayed in real time with B-mode images through a detection pulse that measures the speed of shear waves through the tissue of interest [6].

Pedersen et al. compared testicular stiffness in normal testicular tissue (*n* = 130), testicular microlithiasis (*n* = 99), and GCTs (*n* = 19) using SWE. Their analysis revealed significantly higher mean velocity on SWE in the testicular cancer group compared to those with normal testicular tissue and testicular microlithiasis (*p* < 0.001) [7]. Rocher et al. evaluated the performance of combined B-mode, color doppler, and SWE US in distinguishing between benign and malignant testicular lesions. Their evaluation included 89 focal testicular masses with patients categorized by pathology: malignant tumors (SGCTs, NSGCTs, malignant sex cord Sertoli cell tumor (*n* = 1), and myeloma (*n* = 1)), burned-out tumors, and benign lesions. The following five parameters using SWE were recorded for each testicular lesion: average stiffness with standard deviation (SD), max stiffness, average stiffness/normal testicular tissue stiffness ratio, and max stiffness/normal testicular tissue stiffness ratio. The most relevant conventional US and SWE parameters that best discriminated malignant tumors and burned-out tumors from benign lesions were peripheral vascularization, grouped microliths, and max stiffness/normal testicular tissue stiffness ratio with 92% sensitivity, 96% specificity, 94% accuracy (*p* < 10^−4^), and area under the receiver operating characteristic curve (AUROC) ± 95% confidence interval (CI) of 0.98 ± 0.20. Without the SWE parameters, conventional US had 55% sensitivity, 97% specificity, 74% accuracy (*p* < 10^−4^), and AUROC ± 95% CI of 0.88 ± 0.11. Their group concluded that SWE combined with color doppler US and B-mode US can significantly improve characterization of testicular masses, however, their study was limited by use of a single US operator and subjectivity of the conventional US parameters [7, 8•].

Another method of ultrasonography with potential to help distinguish between benign and testicular GCTs is contrast-enhanced US (CEUS). A bolus of contrast material (microbubbles) is introduced intravenously during simultaneous US of the testicle to demonstrate tissue perfusion. Isidori et al. performed unenhanced and CEUS on 115 patients with non-palpable testicular lesions who subsequently underwent surgical resection. The rapidity of wash-in and washout were the CEUS parameters that best differentiated malignant and benign tumors. Combination of unenhanced and CEUS was highly accurate in diagnosing testicular malignancies (AUROC 0.927 with 95% CI [0.827, 0.981]) [9]. CEUS has yet to be widely validated in the USA and is not routinely used in testicular US.

## Magnetic Resonance Imaging

MRI is not routinely used in the initial evaluation of testicular masses but can be a helpful diagnostic adjunct when US findings are equivocal or if the exact location of an intrascrotal mass is difficult to distinguish. MRI has multiple modalities which are unique in evaluating testicular tumor features. T1- and T2-weighted characteristics can differentiate between fat, soft tissue, and fluid; T1 pre- and post-contrast sequences can assess tumor enhancement; and diffusion-weighted imaging (DWI) can assess water restriction in tissues highlighting neoplastic tissues [10].

Dynamic contrast-enhanced (DCE) MRI obtains information regarding tissue perfusion through the analysis of tissue temporal reaction to the inflow of contrast. More specifically, DCE-MRI provides quantifiable parameters of tissue perfusion, vessel permeability, and microvascular status [11]. Tsili et al. retrospectively studied imaging findings of 44 men who underwent DCE-MRI for intratesticular lesion evaluation. Time-signal intensity curves were plotted for normal testicular tissue and intratesticular lesions. They found that enhancement over time followed one of three curve shapes: type I was a linear increase of contrast enhancement, type II presented as an initial upstroke followed by a plateau or gradual increase, and type III showed an initial stroke-up followed by washout of contrast. Normal testicular tissue enhancement followed a type I curve (100% of cases). Benign intratesticular lesions enhanced with a type II curve (63.6% of benign cases) and testicular carcinomas enhanced heterogeneously with a type III curve (100% of cases) (*p* < 0.001) [12].

DWI is an MRI sequence that provides the functional information regarding tissue diffusion properties. Qualitatively, diffusion is seen on trace images and quantitatively represented on apparent diffusion coefficient (ADC) maps. Malignant tissue, for example, has restricted tissue diffusion due to the increased intracellular proportion of water compared to the extracellular compartment; therefore, it will be bright on trace images and hypointense on the ADC map [13]. A review of 31 scrotal lesions found that the ADC values of normal testis and benign intratesticular lesions were significantly different from testicular malignancies (*p* < 0.05), suggesting that DWI MRI with measurement of ADC may be helpful in characterizing intratesticular masses [14].

Despite these sequences, GCTs can be difficult to distinguish from stromal tumors as they both can appear homogenous with T2-weighted hypointensity and T1 isointesity [15, 16]. Given the paucity of evidence for MRI to differentiate between malignant and benign testicular tumors, its use is recommended for ambiguous cases of testicular lesions, planning for testis-sparing surgery and/or to differentiate between intratesticular and paratesticular lesions [17••, 18].

More specifically, MRI can be used to confirm diagnosis of fatty masses, cystic lesions, and benign solid tumors that are indeterminate on US. Lipomas have high T1 signal intensity on MRI that is specific to adipose tissue but appear non-specifically homogenous and hyperechoic on US [19, 20]. Testicular cysts, which can occasionally appear complex on US, may need an MRI to ensure there are no solid or contrast enhancing components concerning for a cystic testicular neoplasm [21] (Table 1).

## Conclusion

Imaging plays a crucial role in evaluating scrotal masses with ultrasound being the preferred primary imaging modality. The advancement of sonographic technology, CEUS and SWE, has provided clinicians tools that go beyond simple grayscale imaging to help more accurately characterize intratesticular lesions as benign or malignant. When sonography is equivocal, MRI of the scrotum can serve as a supplemental imaging modality to aid in diagnosis and management of an intratesticular lesion.
